# Genomic and Transcriptomic Analysis Provide Insights Into Root Rot Resistance in *Panax notoginseng*

**DOI:** 10.3389/fpls.2021.775019

**Published:** 2021-12-15

**Authors:** Kang Ning, Mengzhi Li, Guangfei Wei, Yuxin Zhou, Guozhuang Zhang, Hao Huai, Fugang Wei, Zhongjian Chen, Yong Wang, Linlin Dong, Shilin Chen

**Affiliations:** ^1^Key Laboratory of Beijing for Identification and Safety Evaluation of Chinese Medicine, Institute of Chinese Materia Medica, China Academy of Chinese Medical Sciences, Beijing, China; ^2^College of Biological and Pharmaceutical Sciences, China Three Gorges University, Yichang, China; ^3^Wenshan Miaoxiang Notoginseng Technology, Co., Ltd., Wenshan, China; ^4^Institute of Sanqi Research, Wenshan University, Wenshan, China

**Keywords:** *P. notoginseng*, biotic stress, root rot, WGCNA, WRKY

## Abstract

*Panax notoginseng* (*Panax notoginseng* (Burk.) F.H. Chen), a plant of high medicinal value, is severely affected by root rot during cultivation. Here, we generated a reference genome of *P. notoginseng*, with a contig N50 size of 241.268 kb, and identified 66 disease-resistance genes (R-genes) as candidate genes for breeding disease-resistant varieties. We then investigated the molecular mechanism underlying the responses of resistant and susceptible *P. notoginseng* genotypes to *Fusarium oxysporum* infection at six time points by RNA-seq. Functional analysis of the genes differentially expressed between the two genotypes indicated that genes involved in the defense response biological process like hormone transduction and plant-pathogen interaction are continuously and highly expressed in resistant genotype during infection. Moreover, salicylic acid and jasmonic acid levels gradually increased during infection in the resistant genotype. Coexpression analysis showed that *PnWRKY22* acts as a hub gene in the defense response of the resistant genotype. Finally, transiently overexpressing *PnWRKY22* increased salicylic acid levels in *P. notoginseng* leaves. Our findings provide a theoretical basis for studying root rot resistance in *P. notoginseng*.

## Introduction

*Panax notoginseng* (Burk.) F.H. Chen is a traditional Chinese herbal medicinal plant in the Araliaceae family that has been used to stop bleeding, reduce swelling, and alleviate pain in China since ancient times. This plant has proven to be useful for treating diseases of the central nervous system and cardiovascular system and is thus widely used as a medicine and health-care product (Wei et al., 2020). However, *P. notoginseng* suffers from many diseases and insect pests during its years of cultivation, including the serious disease root rot. Root rot causes cracking and rotting of the root, the main organ used in medicine, resulting in huge financial losses. *Fusarium oxysporum* is one of the pathogens of *P. notoginseng* root rot (Dong et al., 2016; Yin et al., 2018; Ma et al., 2019). Root rot in *P. notoginseng* is generally controlled with fungicides, which can cause environmental pollution and leave pesticide residues. Only certain soil conditions are suitable for *P. notoginseng* cultivation, the application of fungicides can lead to further reduction of arable soil for *P. notoginseng* cultivation. Thus, breeding disease-resistant genotypes is the most sustainable and eco-friendly measure for disease control in *P. notoginseng*. We recently bred the resistant *P. notoginseng* genotype “Miaoxiangkangqi 1” (MK) (#2016060), which provides an ideal study material for obtaining a deeper understanding of the root rot resistance mechanism in this species. Compared with ZC, the incidence rate of root rot of MK seedlings, 2- and 3-years plants decreased by 83.6, 43.6, and 62%, respectively (Chen Z. et al., 2017; Dong et al., 2017).

Genetic data represent a valuable resource for in-depth studies assessing crop cultivation, secondary metabolite biosynthesis, and genetic improvement. Two versions of the *P. notoginseng* genome have been published in 2017 (Chen W. et al., 2017; Zhang et al., 2017). Recently, three versions of chromosome-level *P. notoginseng* genome with higher continuity were released (Fan et al., 2020; Jiang et al., 2021; Yang et al., 2021). However, as *P. notoginseng* is a highly heterozygous species, its genome sequence still requires further improvement in order to assemble short reads into long contigs and scaffolds. High-quality genome data would serve as a valuable resource for further basic research of disease resistance in *P. notoginseng*.

Plant immune is a complex process. When infected by pathogen, plants initiate disease resistance at the molecular level including signal perception, signal transduction and defense mechanism (Chi et al., 2019). Two types of plant immune mechanisms have been identified: pathogen-associated molecular pattern-triggered immunity (PTI) and effector-triggered immunity (ETI). Surface pattern-recognition receptors (PRRs) recognize pathogen-associated molecular patterns (PAMPs) to induce a basal resistance response (Cui et al., 2015). Disease resistance (R) genes, function in pathogen resistance with the corresponding effectors in a gene-for-gene relationship, are the candidate target gene for breeding disease resistant variety. Most R genes encode proteins containing a nucleotide-binding site (NBS) and leucine-rich repeats (LRRs). NBS domains are involved in signaling, while LRRs are involved in protein-protein interactions (Marone et al., 2013). *RPW8*, another types of R gene, encodes a non-NL R protein that confers broad-spectrum resistance against powdery mildew (Zhong and Cheng, 2016). In contrast to R genes, susceptibility (S) genes facilitate pathogen infection and suppress host immune responses (van Schie and Takken, 2014). Many sugar transporter (*SWEET*) genes function as S genes (Langner et al., 2018). Crops that mutated S gene appear to be more durable than those contain R gene (Deng et al., 2020).

Ca^2+^ serves as a secondary messenger in many signaling processes. The Ca^2+^ signal transduction pathway is triggered when plants recognize a pathogen. Many Ca^2+^-responsive proteins have been identified as critical regulators of plant immunity. Calmodulin (CaM) and calmodulin-like proteins (CMLs) are Ca^2+^ sensors that recognize Ca^2+^ signatures (Boudsocq et al., 2010; Yuan et al., 2018). Several signaling pathways are involved in plant immune mechanisms including MAPK signaling pathway and Plant hormone signal transduction. MAPK cascades are conserved pathways of signal transduction in eukaryotes (Fiil et al., 2009). MAPKs phosphorylate a wide range of target proteins with different roles in plant immunity, which serve as signaling divergence points downstream of immune receptors (Wang et al., 2018). Hormones play important roles in plant responses to biotic stress. The salicylic acid (SA), jasmonic acid (JA), and ethylene signaling pathways respond to biotic stress and subsequently alter the transcript levels of related genes and proteins post-processing (Gupta et al., 2017). SA, in particular, is a major component of systemic acquired resistance (SAR) (Huang et al., 2020). Signals will activate the stress-responsive related genes to defend infection. For example, synthesis of secondary metabolites, protein synthesis and degradation and energy synthesis (Wang et al., 2011). Ultimately, plant survives from the infection of pathogen through a series of defensive responses.

Transcription factors (TFs) are known to be involved in plant immune mechanisms, especially members of the WRKY gene family. WRKY proteins have been implicated in various biotic and abiotic stress responses, including the MAPK signaling pathway, which functions in stress-induced defense responses (Jiang et al., 2017). WRKY proteins contain a 6-amino-acid DNA binding domain (DBD) and a WRKYGQK motif. WRKY TFs bind to the W-boxes (C/T) TGAC(T/C) in their target gene promoters to regulate their expression. For example, in chrysanthemum, WRKY family members function in responses to a variety of phytohormone treatments and to both biotic and abiotic stress (Song et al., 2014). Overexpressing *CmWRKY33.1* resulted in enhanced susceptibility to black spot disease in chrysanthemum (Liu et al., 2020). AtWRKY28 binds to the ISOCHORISMATE SYNTHASE 1 (*ICS1*) promoter to activate SA biosynthesis (van Verk et al., 2011).

In the current study, we assembled a genome of *P. notoginseng* and identified candidate genes for root rot resistant. By using RNA sequencing, we drew the expression pattern map of *P. notoginseng* resist *F. oxysporum* infection and found that the expression of disease-related pathway genes like plant-pathogen interaction, defense response to fungus was more continuous in resistant genotype. Further we revealed that *PnWRKY22* can increased SA levels to improve disease resistance. Thus, our study provides insights into the molecular mechanism underlying root rot resistance in *P. notoginseng* and should facilitate accurate molecular breeding of this crop at the whole-genome level.

## Materials and Methods

### Plant Materials and *Fusarium oxysporum* Inoculation

The susceptible genotype “Common genotype” (ZC) and resistant genotype “Miaoxiangkangqi 1” (MK) were grown in Wenshan, Yunnan Province, China until 2-year-old. A 2 weeks before infection, *P. notoginseng* seedlings were washed, transplanted into plastic square pots (45 cm × 30 cm × 25 cm) at a density of 40 plants/pot, and grown in the greenhouse in Hoagland nutrient solution under a 16 h/8 h light/dark cycle at 25°C and 65–70% relative humidity.

*Fusarium oxysporum* was cultured in liquid potato dextrose medium on a shaker (28°C, 180 rpm) for 5 day in the dark. Subsequently, spore concentrations were determined with a hemocytometer. For treatment, 250 ml of *F. oxysporum* spore suspension (2 × 106 conidia/ml) was added to each pot, and 250 ml liquid potato dextrose medium was added to the control group. Eight hours later, the plants were transplanted to soil and grown under greenhouse conditions. The roots were sampled at 0, 12, 24, 48, 72, and 96 h after inoculation.

### DNA, RNA Extraction and Sequencing

Callus from the “ZC” genotype was used for DNA sequencing to avoid interference from the chloroplast genome. High-quality genomic DNA was extracted from the samples using a Quick DNA Isolation Kit (Waryoung, China). The whole genome was sequenced on the PacBio Sequel System by Anoroad (Beijing, China). A template library was constructed using a SMRTbell Template Prep Kit 1.0 and SMRTbell Damage Repair Kit, annealed with primers, and bound to DNA polymerase using a PacBio DNA/Polymerase Kit and magnetic beads. The samples were loaded onto the PacBio Sequel™ System to read the template sequences.

Root of “MK” and “ZC” at different infected time points were used to extract RNA, all the samples were put into liquid nitrogen immediately and ground into powder under liquid nitrogen condition. Total RNAs were extracted by TPIplant RNA isolation kit (Bioteke, China). The RNA sequencing was performed by Anoroad (Beijing, China).

### Identification of R-Genes in the Whole Genome

Based on a Pfam search, we used the TIR domain (PF01582, PF13676), NB-ARC domain (PF00931), LRR domain (PF00560, PF07723, PF12799, PF13306, PF13516, PF13855, and PF14580), and RPW8 domain (PF05659) to search for R-gene-encoded proteins in the predicted *P. notoginseng* proteome file in genomic data. All the genes were further confirmed to contain the domain in SMART^1^ databases.

### Bioinformatics Analysis

Cutadapt software (^2^ version: cutadapt-1.9) was used to remove the adaptor. And HISAT2 software (^3^ version: hisat2-2.0.4) was used to map reads to the genome. The mapped reads of each sample were assembled using StringTie (^4^ version:stringtie-1.3.4d.Linux_x86_64). The differentially expressed mRNAs were selected with | log2FoldChange| ≥ 1, *q* < 0.05 by R package DESeq2^5^.

K-means cluster analysis and Short Time-series Expression Miner (STEM) analysis were performed in OmicStudio tools^6^. Differentially Expressed Genes (DEGs) identified in different comparison (12, 24, 48, 72, and 96 h with 0 h) in two genotypes were used to perform Short Time-series Expression Miner (STEM) analysis, respectively. The average of the three replicates of each DEGs’ FPKM were analyzed in OmicStudio tools STEM section-STEM clustering method. The data were analyzed under the parameters “Log normalize,” “Bonferronil” and “6 clusters.” DEGs identified in all comparison (12, 24, 48, 72, and 96 h with 0 h) in both genotypes were used to perform K-means clustering analysis. The correlation coefficient as the distance function for K-means clustering (Ernst et al., 2005). The average of the three replicates of each DEGs’ FPKM were analyzed in OmicStudio tools STEM section-K-means. The data were analyzed under the parameters “Log normalize,” “Bonferronil,” and “12 clusters.”

### Measurement of Salicylic Acid and Jasmonic Acid Contents

*Panax notoginseng* roots were flash frozen and ground into powder in liquid nitrogen. Zero point five gram of each sample was extracted with 4 ml methanol:H_2_O:formic acid (15:4:1, v/v/v) by vortexing for 30 s. The mixture was sonicated for 15 min, incubated for 20 min at 20°C, and centrifuged at 10,000 *g* at 4°C for 10 min. The supernatant was transferred to a new centrifuge tube and the precipitate extracted again as described above. The combined supernatants were dried by vacuum evaporation at 45°C, dissolved by 300 μl methanol, and centrifuged at 10,000 *g* at 4°C for 10 min. The upper layer was collected, filtered through a 0.22 μm filter, and transferred to a sample vial. The samples were directly analyzed for phytohormone content by UPLC-MS (Waters Xevo TQ-S, United Kingdom).

### RT-PCR Analysis

Fresh plant tissue was ground into a powder in liquid nitrogen with a mortar and pestle. Total RNA was extracted from the sample with a Quick RNA Isolation Kit (Waryoung, China), and cDNA was synthesized using a FastQuant RT Kit (Tiangen, China). RT-PCR analysis was performed with a SLAN-96P Real-time PCR system (SLAN, China), with each gene analyzed in three biological replicates and three technical replicates using StarLighter SYBR Green qPCR Mix (Forever star, Beijing). The *PnACTIN1* gene was used as an internal reference to normalize gene expression data for *P. notoginseng*. Fold change was calculated using the 2^–ΔΔ^ ^Ct^ method (Livak and Schmittgen, 2001). Details regarding the RT-PCR primers are listed in Supplementary Table 1.

### Phylogenetic Analysis of *PnWRKY22*

The full-length protein sequences of *P. notoginseng* and cucumber (Chen et al., 2020) were listed in the FASTA form and phylogenetic analyzed by MEGA7 software. Sequences were aligned with ClustalW, after which a phylogenetic tree was constructed according to the neighbor-joining method. Reliability of the predicted tree was tested using bootstrapping with 1,000 replicates. Full-length protein sequences for the analyzed sequences are listed in Supplementary Table 2.

### Cloning and Transient Overexpression of *PnWRKY22* in Leaves

The coding region of *PnWRKY22* was cloned by PCR, and the Super1300 vector was linearization by *XbaI* and *KpnI*. The linearized vector and target gene were recombined using a Trelief™ SoSoo Cloning Kit (Tsingke, China). The recombined vector was transformed into *Agrobacterium tumefaciens* strain GV3101. Suspensions contain *A. tumefaciens* were infiltrated into 2-year-old *P. notoginseng* leaves with a needleless syringe. After infiltration, the plants were cultivated 25°C in the light and 18°C in the dark for 2 days. The expression of *PnWRKY22* was detected by qPCR. And the SA content was measure by UPLC-MS. The significance of differences between two groups were analyzed by one-way analysis of variance (ANOVA). *A. tumefaciens* containing empty vector with the same OD_600_ and injection volume was used as a control. Primers used for vector construction are listed in Supplementary Table 1.

### Yeast One-Hybrid Assay

The full-length coding sequence of *PnWRKY22* was cloned into the pB42AD vector. Additionally, three copies of the 30-bp sequence around the W-box *cis-*element of the *PnPAL* promoter were cloned into the pLacZi vector. The reconstructed pB42AD and pLacZi vectors were co-transferred into EGY48 cells. Protein and DNA interactions were assayed on selective medium lacking Trp and Ura and supplemented with 100 mg/μl X-Gal. Primers used for vector construction are provided in Supplementary Table 1.

## Results

### Genome Sequencing and Assembly and Identification of Resistance Genes

*Panax notoginseng* genotype “Common genotype” (ZC) (a widely cultivated genotype) was used to *de novo* genome sequencing and assembly. A 385.28 Gb raw reads were generated, representing approximately 795-fold coverage of the *P. notoginseng* genome and the *de novo* assembly process yielded a draft *P. notoginseng* genome of 1.9 Gb. By using long-read PacBio sequencing, the continuity of our data, with the contig N50 size of 241.268 kb, was significantly improved compared to the two 2017 versions (Supplementary Table 3). We assessed the completeness of the genome and performed annotation with common plant benchmarking universal single-copy orthologs (BUSCOs). Of the 1,375 plant BUSCOs, 1,250 (90.9%) were found in this genome assembly, and 17 plant BUSCOs (1.2%) had fragmented matches (Supplementary Table 4). In total, 45,948 protein-coding genes were predicted, with an average gene length of 4618.92 bp (Table 1). We also predicted 2,382 tRNAs, 29,473 miRNAs, 1,290 snRNAs, and 3,505 rRNAs based on our data (Supplementary Table 5). Using HMMER searches, we identified 66 R-genes in the whole genome, including 17 TIR, 38 NB-ARC, and 11 RPW8 genes (Supplementary Table 6A). Thirty of these R genes are detected in subsequent transcriptome data and these genes are widely expressed during infection, indicating that these genes might be the target genes for breeding disease resistance genotype (Supplementary Table 6B; Supplementary Figure 1A).

**TABLE 1:** Summary of the *P. notoginseng* genome assembly.

Feature	Value
Assembly size (bp)	1,907,700,892
Total number of contigs	11,132
Contig N50 (bp)	241,268
Longest contig	10,492,230
Number of protein coding genes	45,948
Average gene length (bp)	4618.92
Subreads Mean Length (bp)	15703.14
Mean Depth	57.82
Clean Reads	1,517,856,248
Mapped Reads	1,491,800,067
Mapped Reads Rate (%)	98.28
Coverage Rate (%)	91.82

### Generation of Transcriptomic Data of MK and ZC During *Fusarium oxysporum* Infection

Two genotypes with different levels of root rot resistance (the highly resistance genotype MK and the standard genotype ZC) were used as the material. Section of two genotypes’ root showed that periderm of MK is thicker than ZC’s, but the difference was not significant (Supplementary Figures 2A–C). It suggested that the disease resistance molecular mechanism might play a more important role in MK disease-resistant.

To further explore the mechanism of root rot resistance in *P. notoginseng*, we performed comparative transcriptome analysis of two genotypes at six time points (0, 12, 24, 48, 72, and 96 h) during infection. Total of 1,564,500,538 clean reads were generated from 36 samples (three replicates of both genotypes sampled at each time point), with each sample producing a minimum of 39,102,454 clean reads (Supplementary Table 7). Following assembly, we obtained a set of 181,716 unigene sequences with a mean length 679.55 nucleotides (nt). The transcript abundances (as estimated from the number of fragments per kilobase of transcript per million mapped reads [FPKM]) were highly correlated (Pearson’s correlation coefficients higher than 0.81) between replicate samples (Supplementary Table 8). The root of ZC is sticky in the later stages of infection (48∼96 h). while the surface of MK root is smooth (Supplementary Figures 2D,E). These results indicate that the two genotypes show different patterns of defense gene expression during infection and MK is more resistant to root rot.

### Analysis of the Differentially Expressed Genes That Identified by Different Comparison

**FIGURE 1:**
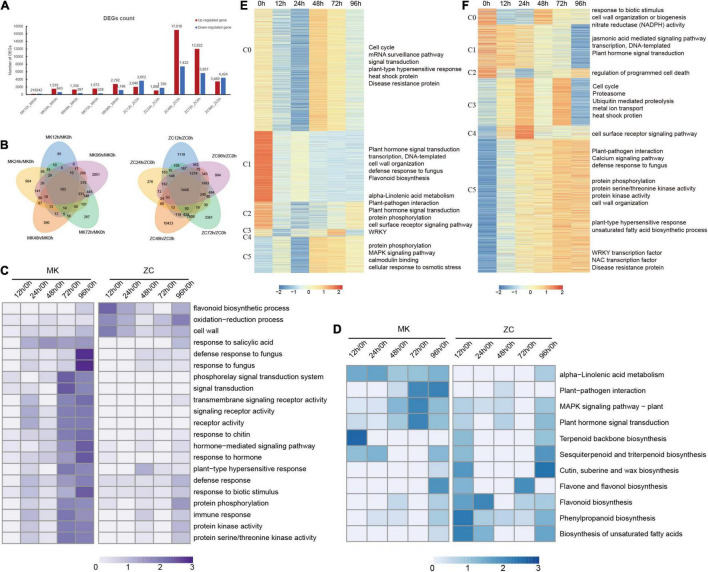
Statistical and functional analysis of differentially expressed genes (DEGs) in MK and ZC during infection. **(A)** Number of upregulated and downregulated DEGs at different time points compared to 0 h in MK and ZC. **(B)** Venn diagrams of the different comparison of MK and ZC. **(C,D)** GO term **(C)** and KEGG pathway **(D)** enrichment of DEGs in pairwise comparisons in MK and ZC. Z-score value of normalized –log_10_ (*p*-value) of each item was used to draw heatmap. **(E,F)** gene expression pattern and function of DEGs in ZC **(E)** and MK **(F)**. Z-score value of normalized FPKM of gene was used to draw heatmap. GO and KEGG enriched function item are showed on the right.

By comparing later time points (five total: 12, 24, 48, 72, and 96 h) with the uninfected control (0 h), 6,145 and 31,938 unique DEGs are identified in MK and ZC, respectively. In MK, the number of DEGs gradually increased during the course of infection, whereas in ZC, the number of DEGs decreased at the 12 and 24 h time points, peaked after 48 h, and then gradually decreased toward the 72 h and then 96 h time points (Figure 1A). Kyoto Encyclopedia of Genes and Genomes (KEGG) analysis revealed that DEGs identified in MK are enriched in metabolic pathways, like secondary metabolites, linoleic acid and alpha-Linolenic acid metabolism. They are also enriched in plant-pathogen interaction, plant hormone signal transduction pathway. Signal transduction genes, sensory system and environmental adaptation categories have large proportion than other items in MK (Supplementary Figures 3A,B). While, DEGs identified in ZC are enriched in ribosome, phenylpropanoid and flavonoid biosynthesis (Supplementary Figures 3C,D). In MK, there are few common DEGs among 5 comparisons and a large number of uniquely expressed DEGs were detected at each stage (Figure 1B). These results suggest that unique defense mechanisms are employed at each stage of infection and signal transduction play an important role in MK defense against *F. oxysporum*.

Gene ontology (GO) analysis showed that genes in the categories flavonoid biosynthetic process, oxidation–reduction process and cell wall are enriched in ZC. While, signal transduction, transmembrane signaling receptor activity and hormone-mediated signaling pathway are enriched in MK infection. Defense response, response to biotic stimulus and protein phosphorylation pathway were enriched in both genotypes and particularly enriched in MK later infection stages, indicating that these processes play important roles in defense in this MK (Figure 1C). KEGG analysis revealed that the MAPK signaling pathway, plant hormone signal transduction, and plant-pathogen interaction were enriched during infection in MK. Genes involved in alpha-linolenic acid metabolism were also highly expressed in MK during infection. By contrast, the DEGs in ZC were enriched in the pathways phenylpropanoid biosynthesis, flavonoid biosynthesis, and biosynthesis of unsaturated fatty acids (Figure 1D).

We also perform GO analysis of the upregulated genes in MK, finding that genes in the categories cell recognition, transcription regulator activity, calmodulin binding, signal transduction, response to stimulus, and transmembrane signaling receptor activity were enriched in this genotype (Supplementary Figure 4). A directed acyclic graph (DAG) showed that response to stimulate process genes are persistently enriched after 12 h (Supplementary Figure 5). By contrast, the DEGs in ZC were enriched in the metabolic process (Supplementary Figures 3, 6). These results indicate that response to stimulus, hormone signal transduction, and transcriptional regulation play critical roles in the defense response of *P. notoginseng* and that MK has a clear advantage in these processes.

By using Short Time-series Expression Miner (STEM) method, DEGs identified in MK and ZC were classified into 6 cluster, respectively (Figures 1E,F; Supplementary Table 9). Nearly half of DEGs (C5) in MK are gradually increased during the infection and these genes are enriched in biotic stress defense related pathways including plant-pathogen interaction, defense response to fungus and plant-type hypersensitive response. Calcium signaling was served as second messengers in plant biotic stress (Aldon et al., 2018), CBL-interacting protein kinase (CIPK) family members participate in in ions transport and stress responses (Batistic and Kudla, 2004). Two family members (*PnCIPK12*, *PnCIPK4*) were highly expressed in the late stage of MK infection. Post-Translational Modifications (PTMs), introducing functional group to the protein to change protein function, is reported to be involved in biotic stress. Protein phosphorylation, permitting plant to have a rapid response to stimulation, highly involved in signal transduction (Peck, 2003). Protein phosphorylation biological process related genes are highly expressed in the later stages of MK infection. Meanwhile stress-related transcription factor WRKY, NAC and disease resistance genes are also increasing expressed during MK infection (C0-C4). Other disease-resistant process like cell surface receptor signaling, plant hormone signal transduction and proteasome were also highly expressed during MK infection. Although, DEGs in ZC are also enriched in defense response like plant-pathogen interaction, protein phosphorylation and MAPK signaling pathway, but genes involved in these pathways didn’t successively express through infection. These results suggested that *P. notoginseng* anti-root rot is a complex process involving signal perception, signal transduction, defense response and transcriptional regulation. And MK showed an advantage in continuity of these processes than ZC, leading a better disease resistance.

**FIGURE 2:**
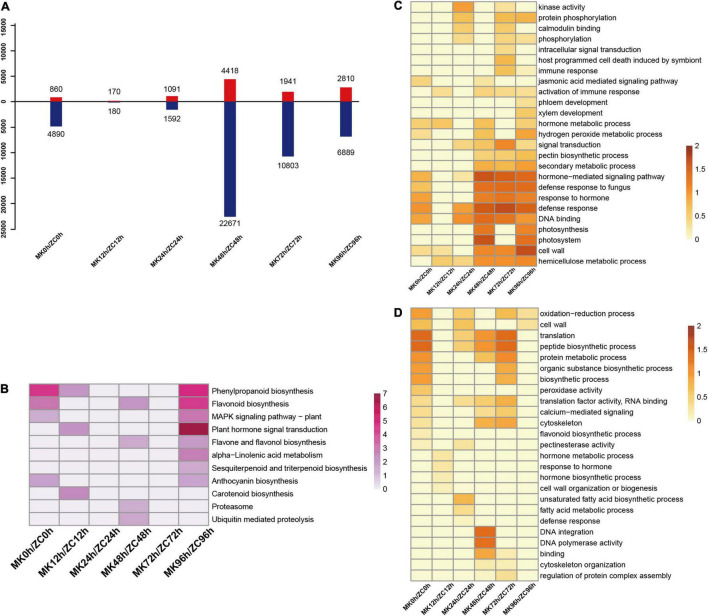
Statistical and functional analysis of DEGs between MK and ZC. **(A)** Number of upregulated and downregulated DEGs at the same time points between MK and ZC. **(B)** KEGG term enrichment of DEGs from same time points between MK and ZC. **(C,D)** GO term enrichment of upregulated **(C)** and downregulated **(D)** DEGs in pairwise comparisons. The data were calculated by –log_10_ (*p*-value).

We also compared the transcriptomes of the two genotypes at the same time points and identified 36,683 DEGs (Figure 2A) KEGG analysis revealed that there is a significant difference in MAPK signaling pathway, Plant hormone signal transduction, and secondary metabolite biosynthesis including phenylpropanoid, flavone, and sesquiterpenoid between the two genotypes at 96 h of infection (Figure 2B). GO analysis showed that defense response, DNA binding, hormone-mediated signaling pathway, and cell wall related genes were highly expressed in MK after 48 h (Figures 2C,D). Thus, plant hormones, the MAPK signaling pathway, and defense mechanisms might have important roles in the disease resistance of MK.

### Identification of the Expression Patterns of the Differentially Expressed Genes by K-Means Clustering

**FIGURE 3:**
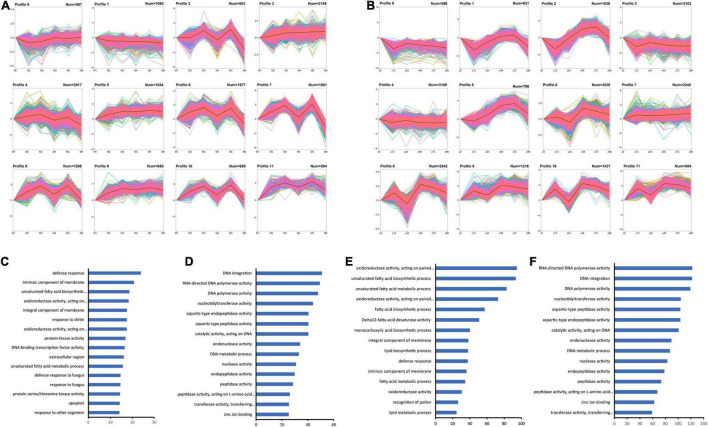
*K*-means clustering of DEGs in MK and ZC. **(A,B)** Twelve clusters of MK **(A)** and ZC **(B)** DEGs based on FPKM values. **(C–F)** GO term enrichment of the DEGs in MK profile 3 **(C)**, MK profile 7 **(D)**, ZC profile 7 **(E)**, and ZC profile 10 **(F)**. The data were calculated by –log_10_ (*p*-value).

To identify the expression patterns of the DEGs in both genotypes of *P. notoginseng* during *F. oxysporum* infection, we performed two independent *K*-means clustering tests for the 36,023 DEGs in the two genotypes and generated 12 optimal clusters for each genotype (Figures 3A,B; Supplementary Table 9). Some profiles share similar patterns between the two genotypes. For example, genes in profiles 3 of MK and profile 7 of ZC showed increasing expression during the course of infection and are functioned in defense response, and unsaturated fatty acid biosynthetic process (Figures 3C,E). Genes that enriched in DNA integration, DNA metabolic process, RNA-directed DNA polymerase activity, and peptidase activity share different expression patterns in two genotypes (Figures 3D,F). These results indicate that the plants execute biosynthetic processes to defend against infection, but the two genotypes respond at different time points.

### Hormones Play Important Roles in the Defense Response of MK

**FIGURE 4:**
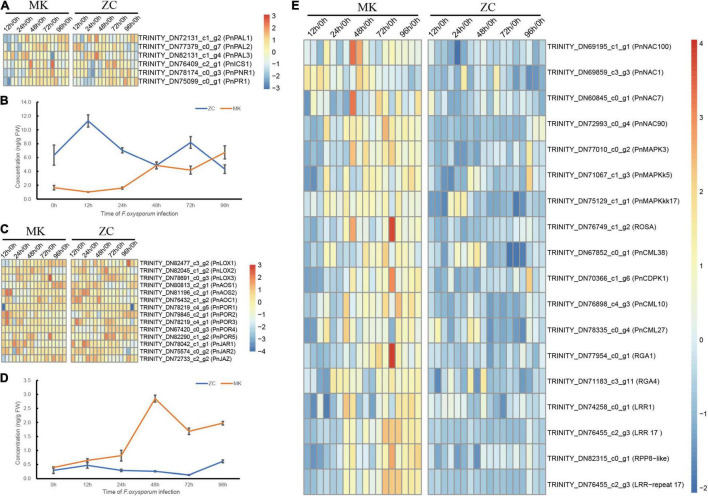
Hormone levels and candidate genes expression during *F. oxysporum* infection in MK and ZC. **(A,B)** Effects of *F. oxysporum* infection on salicylic acid (SA), Heatmaps of SA biosynthesis and signal transduction-related gene expression **(A)** and concentrations of SA during infection **(B)** in MK and ZC. **(C,D)** Effects of *F. oxysporum* infection on jasmonic acid (JA), Heatmaps of JA biosynthesis and signal transduction-related gene expression **(C)** and concentrations of JA during infection **(D)** in MK and ZC. **(E)** Heatmap of the candidate genes that involved in *P. notoginseng* root rot resistance.

Function analysis of DEGs revealed that SA and JA signal transduction genes such as *PNR1*, *PR1*, and *JAZ* were highly expressed during the later stages of *F. oxysporum* infection (Figures 4A,C). To demonstrate the role of SA and JA in defend against infection, we analyzed contents of JA and SA in both *P. notoginseng* genotypes at different time points. In MK, both SA and JA contents gradually increased during the infection process. In ZC, by contrast, the SA content in decreased during this process, but the JA content did not change significantly (Figures 4B,D). We measured the expression of JA- and SA-metabolism-related genes based on the RNA-seq data. Three *isochorismate synthase* (*ICS*) and three *phenylalanine ammonia lyase* (*PAL*) genes, which are major genes involved in the ICS and PAL pathways, respectively, leading to SA biosynthesis, were identified in the data set. The expression of these genes gradually increased during infection in both genotypes. *PnLOX1*, *PnAOS1*, and *PnAOC1* expression gradually increased in MK during infection (Figures 4A,C). These results suggested that SA and JA play important roles in MK defend against infection.

The MAPK pathway, Ca^2+^ signaling, respiratory burst oxidases (ROS), R genes, and NAC family members play important roles in plant defense three MAPK-related genes, one respiratory burst oxidase gene, four Ca^2+^ signaling genes, four NAC gene family members, and six R genes as candidate genes with likely functions in MK resistance. These genes were highly expressed during MK infection (Figure 4E). We also identified sugar transporter (*SWEET*) genes in our transcriptome data set, some of which were highly expressed in the susceptible genotype; these genes might represent S genes (Supplementary Figure 1B). We chose several genes to preform Q-PCR, and Q-PCR results are consistent with RNA-seq (Supplementary Figure 7).

### Weighted Correlation Network Analysis (WGCNA) Shows That *PnWRKY22* Acts as a Hub Gene in MK Infection

**FIGURE 5:**
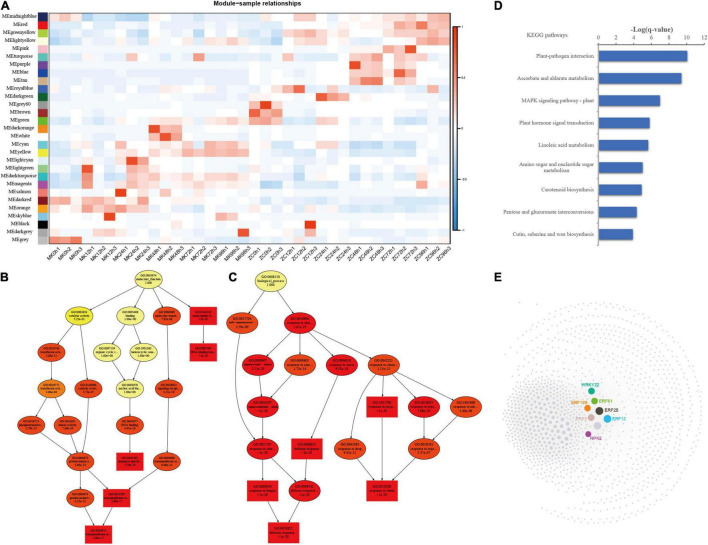
WGCNA of the transcripts and functional analysis of genes in the yellow module. **(A)** Module-sample relationship. **(B,C)** Directed acyclic graph (DAG) of genes in the yellow module in the Biological Process **(D)** and Molecular Function **(E)** categories. **(D)** KEGG analysis of the genes involved in the yellow module. The data were calculated by –log_10_ (*p*-value). **(E)** Coexpression network of the genes clustered in yellow module.

To identify the gene regulatory network of *P. notoginseng* in response to *F. oxysporum* infection, we performed WGCNA using the RNA sequencing profiles. A total of 38,562 genes were subjected to WGCNA, ultimately revealing 29 gene coexpression modules (Figure 5A; Supplementary Figure 8A). The number of genes in these modules ranged from 31 to 15,247. Different modules were correlated with different infection time points in the two genotypes. The orange/dark red modules were correlated with the 12–24 h after infection time points, the dark orange/white and light cyan modules were correlated with the 48 h after MK infection time points, and the cyan/yellow modules were correlated with the 72–96 h after MK infection time point. The dark turquoise and yellow modules were correlated with the later infection period (24–96 h), and the royal blue, dark green/turquoise/purple/blue/tan, and red/green yellow/pink modules were correlated with different time points of ZC infection.

We focused on the yellow module, which was correlated with the later infection stages (48–94 h) of MK infection, for further analysis. DAG showed that genes in the yellow module are involved in transcription regulator activity and protein kinase activity and are enriched in defense response and response to hormones, biotic stimulus, and fungus biological pathways (Figures 5B–D). KEGG analysis showed that genes related to plant-pathogen interaction, the MAPK signaling pathway, and plant hormone signal transduction were enriched in the yellow module (Figure 5D). Five ERF genes, one WRKY gene, and *RPK2* play important roles in this module. These genes were highly expressed in MK at 72–96 h after infection (Figure 5E; Supplementary Figure 8B). These results suggest that the plant-pathogen interaction, MAPK signaling, and plant hormone signal transduction pathways play important roles in the defense response in MK and that these genes are critical for this response.

### Characterization of *PnWRKY22* in *Panax notoginseng*

**FIGURE 6:**
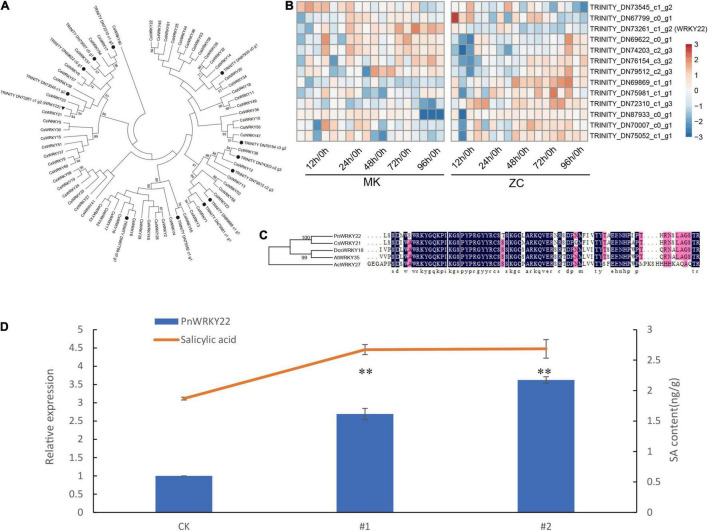
Characterization and functional analysis of *PnWRKY22*. **(A)** Phylogenetic tree analysis of WRKYs identified in *P. notoginseng* and cucumber. **(B)** Expressions of *P. notoginseng* WRKYs in two genotypes during infection. **(C)** Sequence alignment of *PnWRKY22* and other WRKY. **(D)** Relative expression levels of *PnWRKY22* and SA content in WT and 2 OE lines. Data are expressed as the means ± SD, *n* = 3. The asterisks denote significant differences according to one-way analysis of variance (ANOVA) (^**^P < 0.01).

*PnWRKY22*, which acts as a hub gene in the yellow module, might play an important role in the defense response in MK. To investigate this notion, we functional analyzed the *PnWRKY22*. Phylogenetic analysis of *P. notoginseng* WRKY family members with *Cucumis sativus* L showed that PnWRKY22 is clustered with CsWRKY21, which belonging to II e subgroup (Figures 6A,C). And most of *P. notoginseng* WRKY family members are highly expressed in the later stages of infection (Figure 6B).

Then, we transiently overexpressed *PnWRKY22* in the leaves of MK. The SA content was higher in the OE (overexpression) lines than in the wild type (WT; empty vector), suggesting that *PnWRKY22* might regulate SA biosynthesis to promote disease resistance (Figure 6D). Only the *PAL1* promoter sequence was identified in our genome data, and two W-boxes, which WRKY proteins might bind to, were found in its promoter. But, PnWRKY22 can’t bind to these W-boxes by yeast-one hybrid assay (Supplementary Figure 9). These results indicate that *PnWRKY22* can promotes SA biosynthesis, but not through regulating the expression of *PnPAL1* to promote SA biosynthesis.

## Discussion

Root rot seriously affects the cultivation of *P. notoginseng*, but the molecular mechanisms underlying *P. notoginseng* defense against root rot are poorly understood. We previously determined that *F. oxysporum* is one of the pathogens responsible for root rot in *P. notoginseng* and bred MK, a resistant genotype (Dong et al., 2016, 2017; Chen Z. et al., 2017). Therefore, we compared the transcriptomes of the susceptible and resistant genotypes during *F. oxysporum* infection, which enabled us to identify a series of *P. notoginseng* genes that respond to *F. oxysporum* infection.

Morphological characteristics such as thicker epidermis, secreting sticky substances, and covering wax can reduce microbial invasion (Wu et al., 2017; Lewandowska et al., 2020; Yan et al., 2020; Zhang et al., 2020). When pathogen breaks through the mechanically defense, plant immune will initiate to further restrict the colonization and entry of these microbes (Deng et al., 2020; Zhang et al., 2020). There is no significantly difference between two genotypes’ thickness of periderm, suggesting that MK has advantages in biotic stress resistance at molecular level. RNA-seq can identify the expressed genes at the time point. ZC contains more DEGs than MK during infection, suggesting that MK underwent few biological changes, while ZC underwent huge changes leading to a large number of gene changes. Transcriptome data showed that MK has a more complete regulatory network for biotic stress resistance, while ZC might have defect in signal perception and transduction (Figure 1). For example, (1) DEGs identified in MK are uniquely enriched in plant-pathogen interaction and signal transduction and environmental adaptation related genes were overrepresented in the MK DEGs. (2) Biotic defense related items like defense response to fungus, signal transduction and signaling receptor activity were highly enriched during MK infection, while these items were not enriched during ZC infection. (3) STEM analysis revealed that signal perception, signal transduction, defense mechanism related genes and transcriptional factors are continuous express during MK infection. Metabolic pathways like flavonoid and phenylpropanoid biosynthesis were consistently expressed during ZC infection, suggesting that ZC may inhibit infection by synthesizing secondary metabolites (Wang et al., 2011; Meena et al., 2017). (4) Overexpressing *PnWRKY22*, a hub gene in the yellow module, led to an increase in SA content. However, the expression of *PnWRKY22* did not show a significant change during ZC infection. Transcriptional factors are regulated by signal transduction like MAPK pathway to regulate stress-responsive genes (Pandey and Somssich, 2009; Adachi et al., 2015; Chi et al., 2019). Thus, ZC might be defective in signal transduction, leading to failure to regulate downstream stress-resistant genes.

Plant hormones, especially SA and JA, play important roles in disease resistance in plants (Dong, 1998; Alvarez, 2000; Kumar, 2014; Zhu et al., 2014). SA and JA contents gradually increased in resistant genotype MK during infection, whereas SA contents decreased in the susceptible genotype ZC. However, the average SA content of the susceptible genotype ZC was higher than that of the resistant genotype MK (Figures 4B,D), suggesting that the variation in hormone content is more important than the absolute content of the hormone for plant disease resistance. Meanwhile, SA and JA signal transduction genes were active in the resistant genotype during infection (Figures 4A,C). Thus, after infection, the synthesis of SA and JA began to increase, and SA and JA signal transduction process increased to initiate defense process. It has been reported that WRKY can regulate salicylic acid synthesis (van Verk et al., 2011). Compared with WT, the SA content of the two OE lines increased. The expression levels of *PnWRKY22* were different in the two OE lines, but there was no significant difference in SA content. This may be due to the existence of feedback regulation in plants, and WRKY could not promote SA synthesis without limit (Liu et al., 2020). It suggests that plants defense pathogen infection in many ways.

*Panax notoginseng* is a species used in traditional Chinese medicine with high medicinal value. As the demand for this medicinal plant increases, so does the demand for superior genotypes, such as those with high medicinal compound contents, disease resistance, and wide adaptability of soil type. As *P. notoginseng* is a perennial plant, traditional breeding of this medicinal plant is not highly efficient. Thus, the application of new techniques, such as molecular-assisted breeding, to breed new varieties of *P. notoginseng* will facilitate the development of the industry. The botanical characteristics of *P. notoginseng* have hampered basic research in this crop. The high heterozygosity of *P. notoginseng* has led to poor quality of genomic data, limiting basic research on this plant. With improvements in sequencing technology, the genomic quality of *P. notoginseng* has gradually increased. By using PacBio long reads technology, continuity of recent genome versions has significantly improved. Meanwhile, material used for sequencing also have an impact on the quality of the genome (Chen W. et al., 2017; Zhang et al., 2017; Fan et al., 2020; Jiang et al., 2021; Yang et al., 2021). In the future, more homozygous varieties and more advanced sequencing techniques will further enhance genomic quality. Although *P. notoginseng* genome has been significantly improved. As mentioned above, poor genome quality has prevented the identification of the promoter regions of *ICS*, *PAL*, or other SA biosynthesis pathway genes in this study. Technological innovations should result in a high-quality genome for this species in future. Furthermore, transgenic systems for *P. notoginseng* are not well developed, and it is difficult to obtain transgenic plants. Meanwhile, it’s more convincing to overexpress the *PnWRKY22* in the root, but *P. notoginseng* is known for its hard roots, which make it impossible to inject suspensions in the roots. Thus, in the current study, we transiently overexpressed *PnWRKY22* in leaves to verify its function. In the future, advances in research and the development of a gene functional verification systems should enable people to make better use of this herbal medicine.

## Data Availability Statement

The datasets presented in this study can be found in online repositories. The names of the repository/repositories and accession number(s) can be found in the article/Supplementary Material.

## Author Contributions

KN, ML, and LD designed the research. KN, ML, GW, YZ, and HH performed the research. ML, FW, ZC, and YW provided and cultivated the materials. KN, ML, and GZ analyzed the data. KN, ML, and GW wrote the manuscript. LD and SC revised the manuscript. All authors contributed to the article and approved the submitted version.

## Conflict of Interest

FW is employed by Wenshan Miaoxiang Notoginseng Technology, Co., Ltd. The remaining authors declare that the research was conducted in the absence of any commercial or financial relationships that could be construed as a potential conflict of interest.

## Publisher’s Note

All claims expressed in this article are solely those of the authors and do not necessarily represent those of their affiliated organizations, or those of the publisher, the editors and the reviewers. Any product that may be evaluated in this article, or claim that may be made by its manufacturer, is not guaranteed or endorsed by the publisher.

## Footnotes

1
http://smart.embl-heidelberg.de/

2
https://cutadapt.readthedocs.io/en/stable/

3
https://daehwankimlab.github.io/hisat2/

4
http://ccb.jhu.edu/software/stringtie/

5
https://www.bioconductor.org/packages/release/bioc/html/DESeq2.html

6
https://www.omicstudio.cn/tool
