# Supplementary material for: Genomic and Transcriptomic Analysis Provide Insights Into Root Rot Resistance in Panax notoginseng
Source: Front Plant Sci. 2021 Dec 15;12:775019. doi: 10.3389/fpls.2021.775019 (PMC8714957; doi:10.3389/fpls.2021.775019)
Supplement: Supplementary file 10 [file Presentation_1.pdf]

Supplementary Material

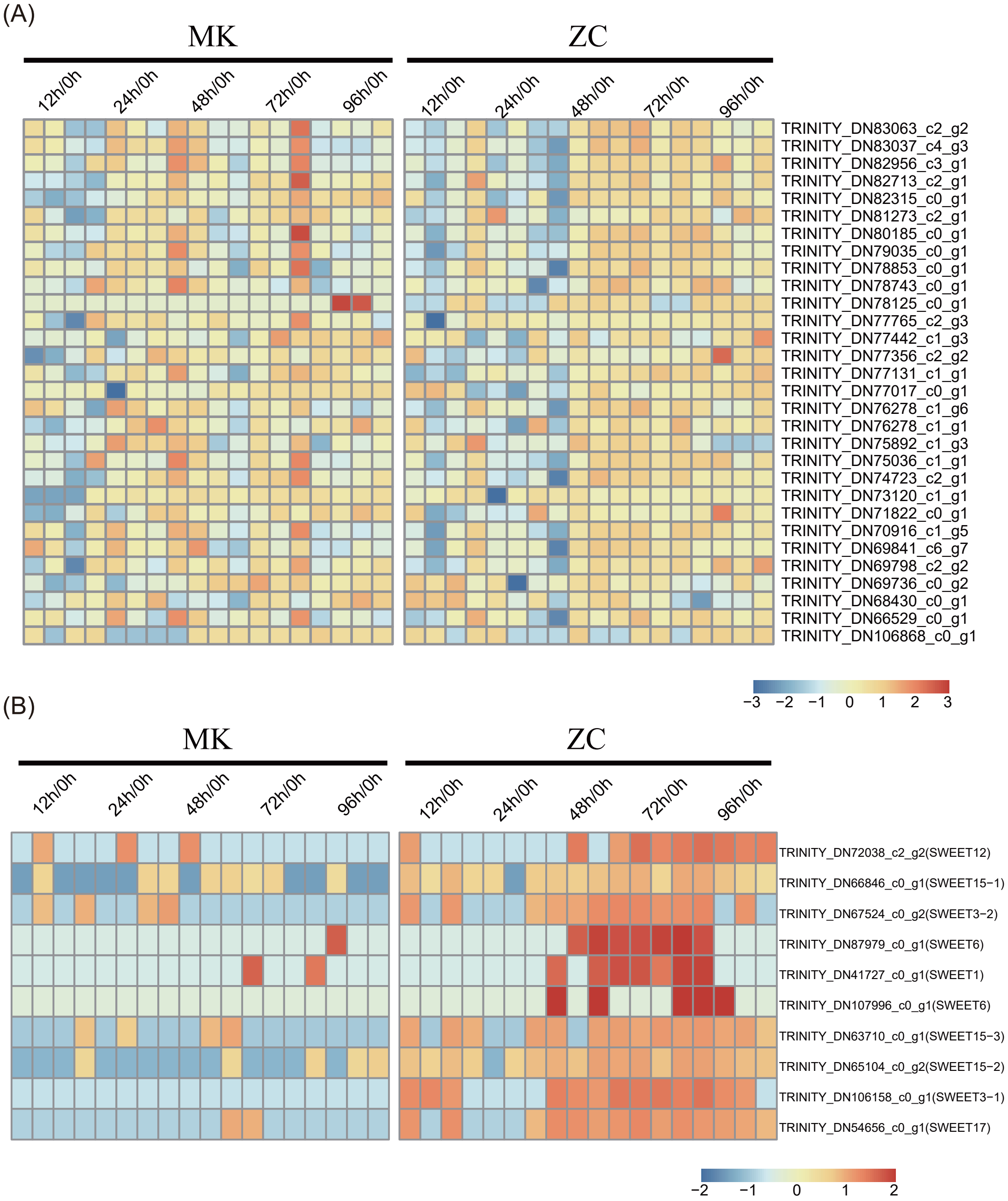

Supplementary Figure 1 | Expression levels of the R genes and S genes during infection.

R genes(**A**) and S genes(**B**) expression pattern during two genotypes infection.

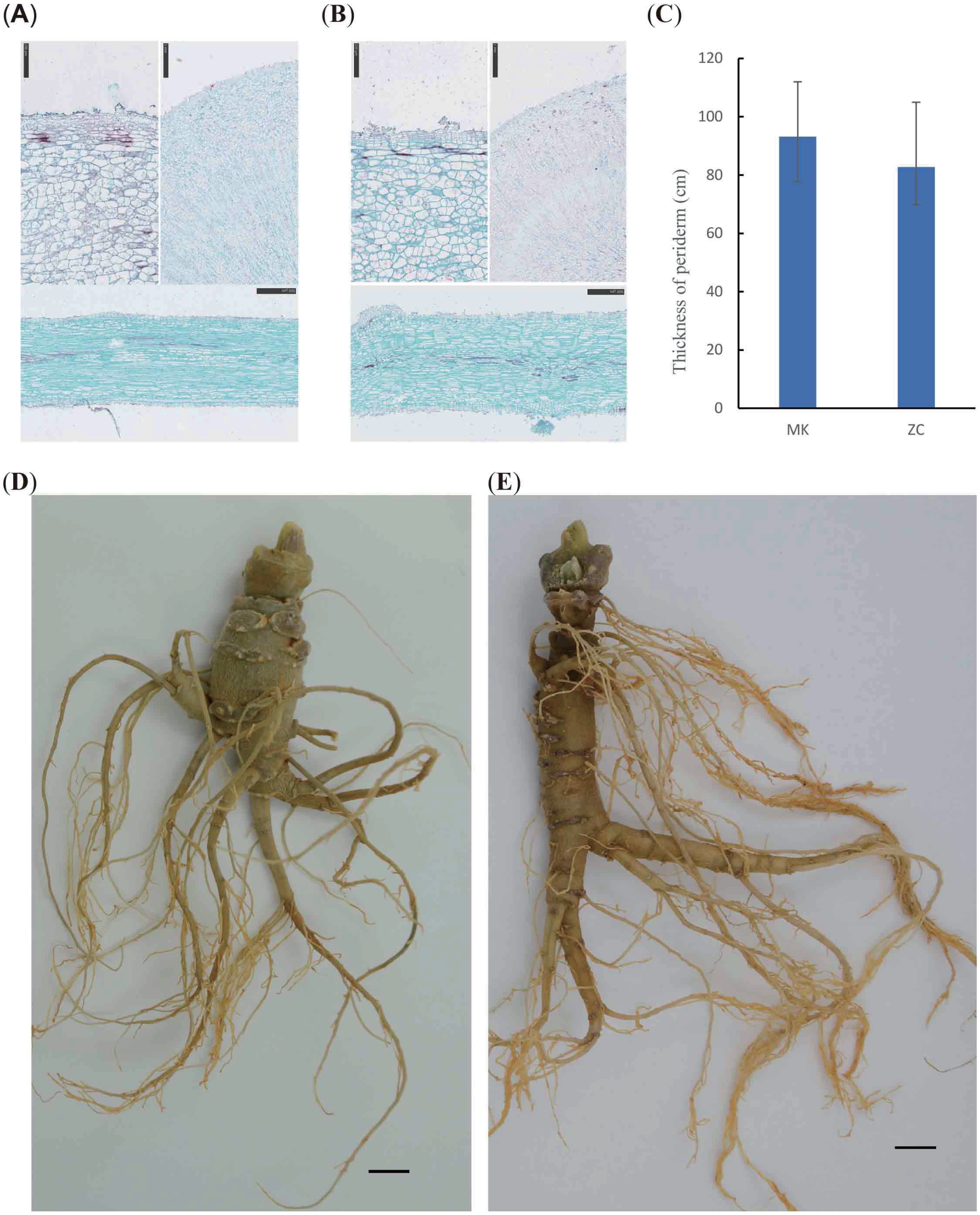

Supplementary Figure 2 | Characterization of two *P. notoginseng* genotypes.

(**A**, **B**) Section of MK (**A**) and ZC (**B**) root. (**C**) Thickness of two *P. notoginseng* genotypes periderm. No significant differences between two genotypes according to one-way analysis of variance (ANOVA) (p= 0.107). (**D**, **E**) Phenotype of MK (**D**) and ZC (**E**) root after 96h infection.

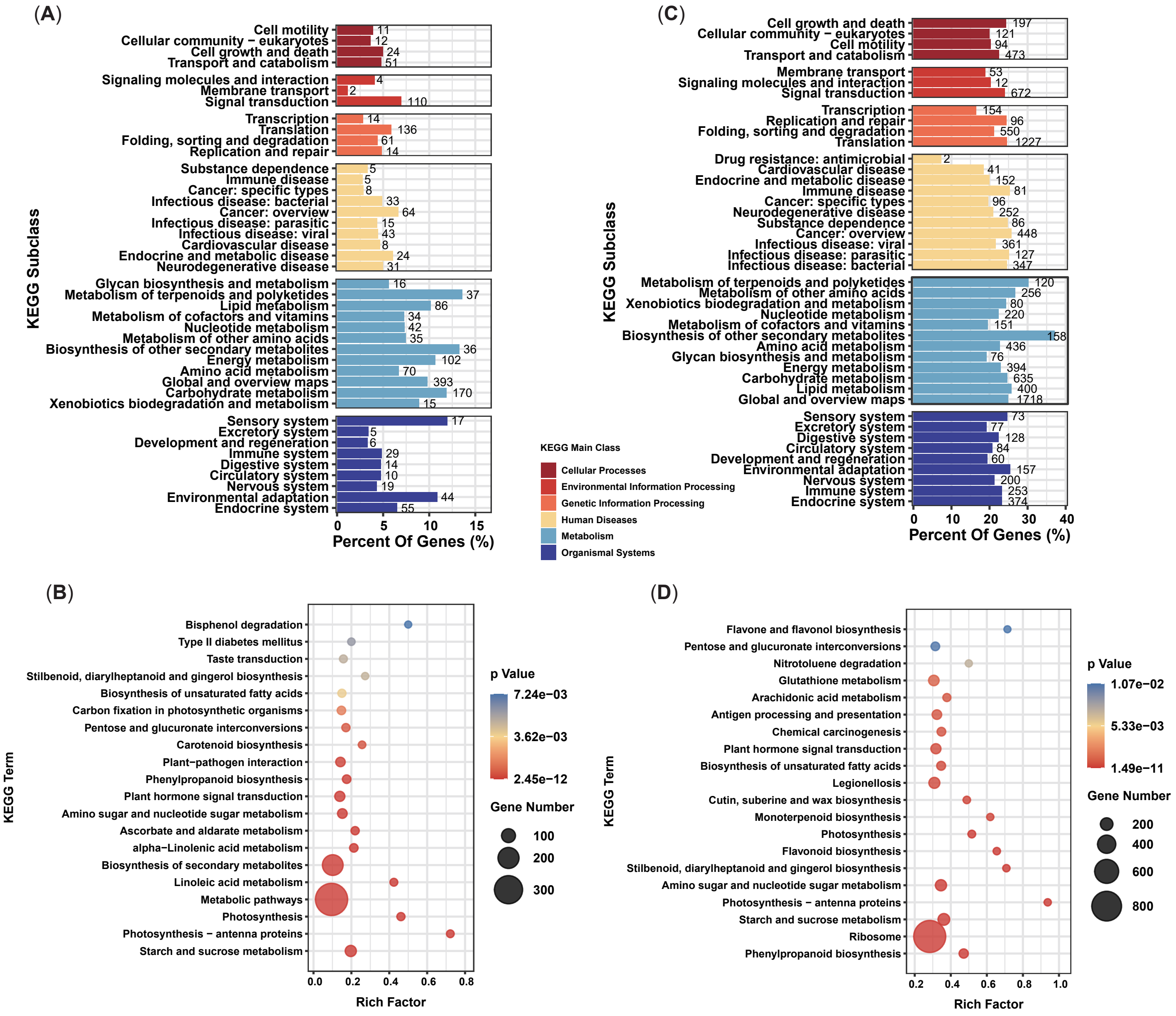

Supplementary Figure 3 | KEGG analysis of DEGs identified in MK and ZC

(**A, C**) KEGG enrichment bar polt of DEGs in MK(**A**) and ZC(**C**). (**B, D**) KEGG enrichment scatter polt of MK(**B**) and ZC(**D**).

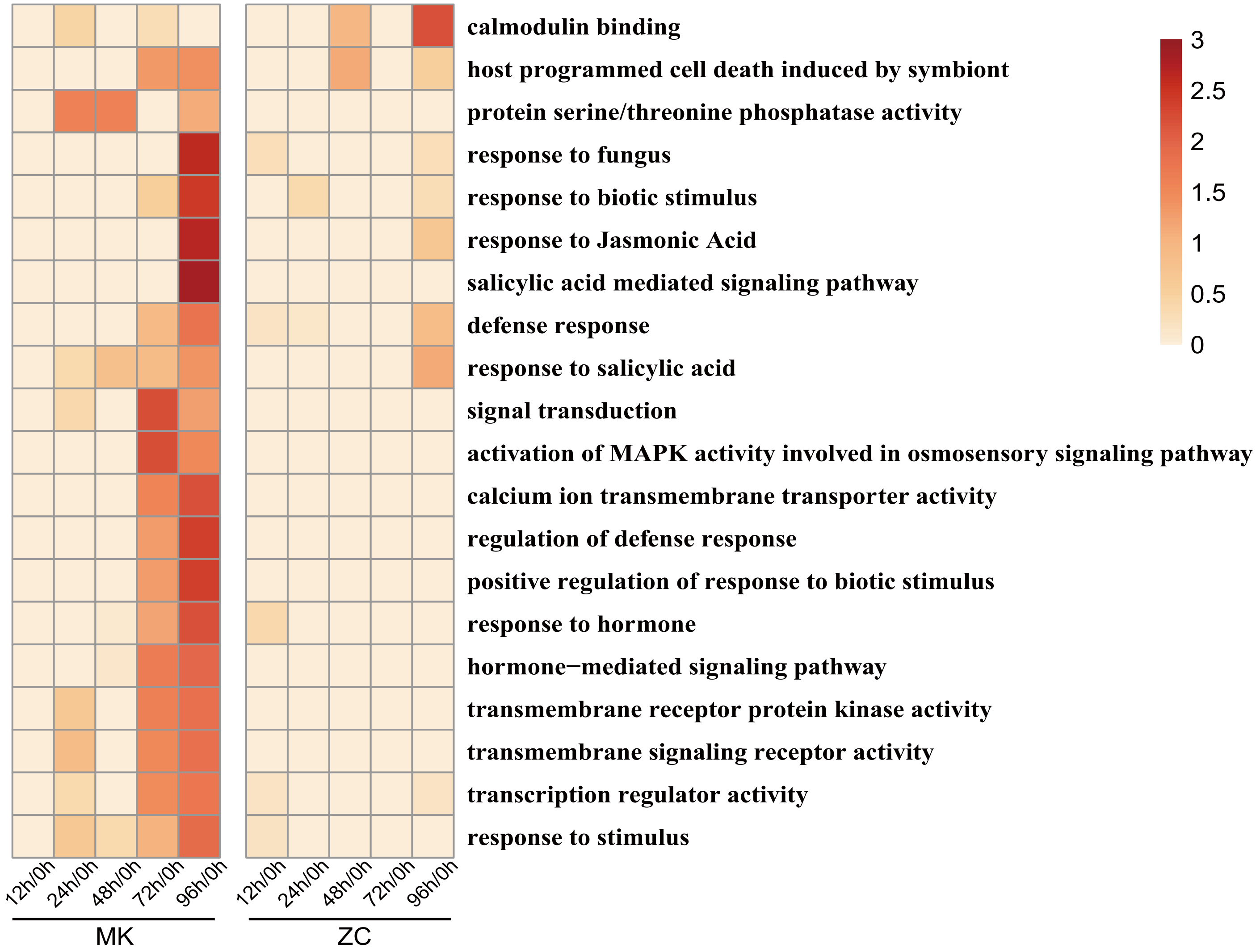

Supplementary Figure 4 | Enrichment of GO functional categories in the upregulated and downregulated DEGs

GO term enrichment of upregulated DEGs in pairwise comparisons. The data were calculated by -log10(*p-value*).

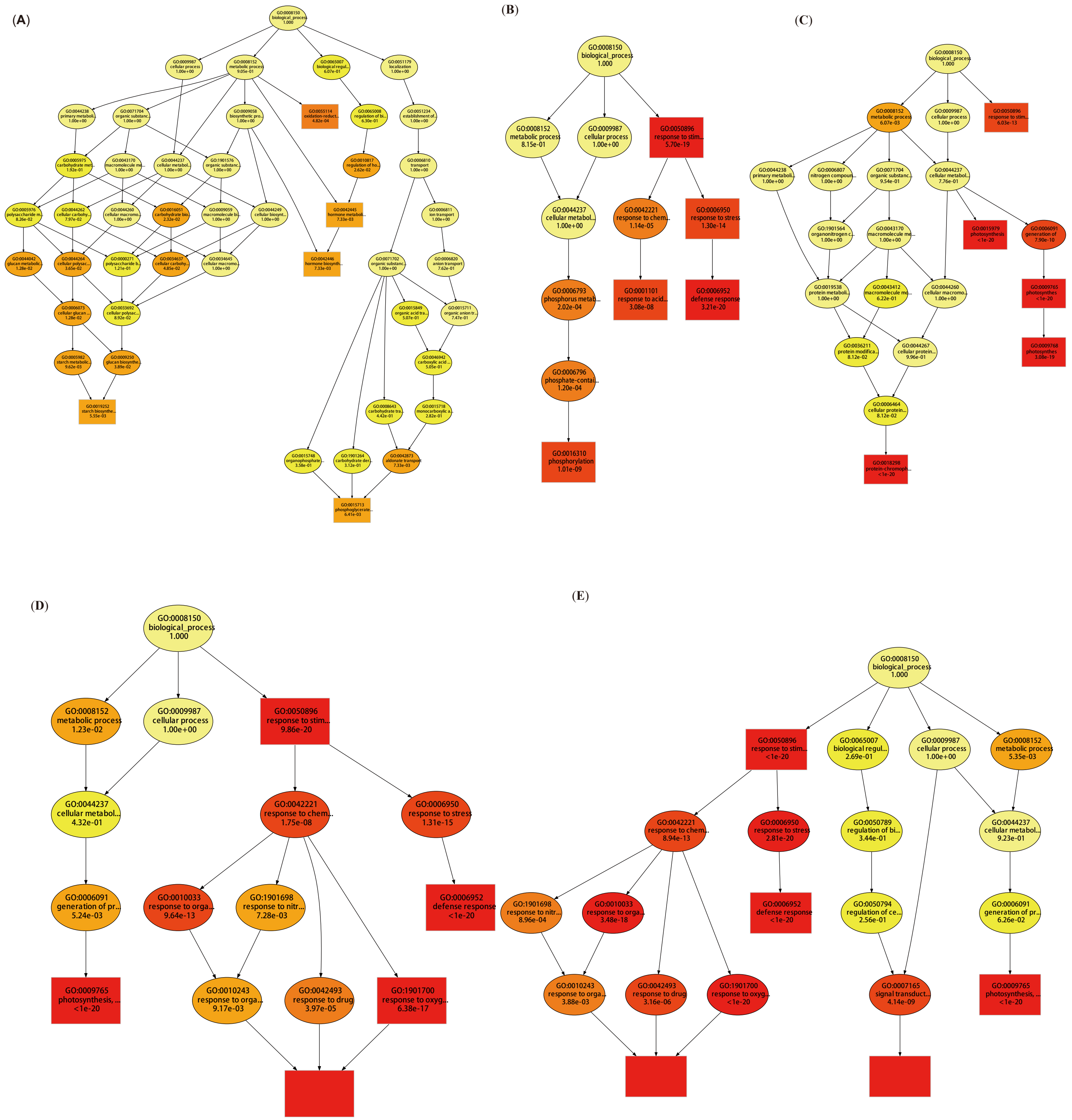

Supplementary Figure 5 | DAG of the DEGs in different comparisons in MK.
(**A-E**) DAG of the DEGs identified in comparisons of 12h, 24h, 48h, 72h, and 96h with 0h in MK, respectively.

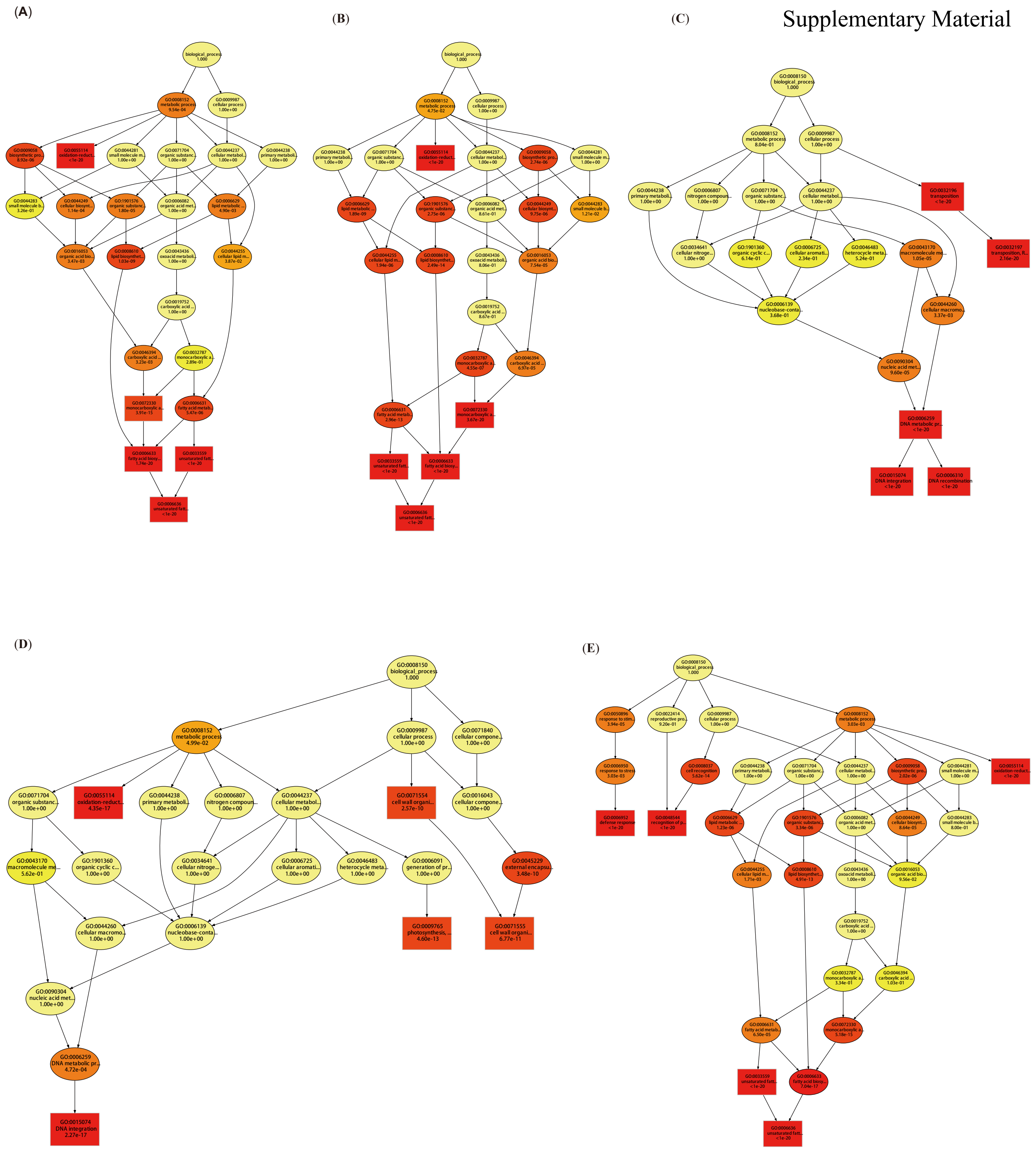

Supplementary Figure 6 | DAG of the DEGs in different comparisons in ZC.
(**A-E**) DAG of the DEGs identified in comparisons of 12h, 24h, 48h, 72h, and 96h with 0h in ZC, respectively.

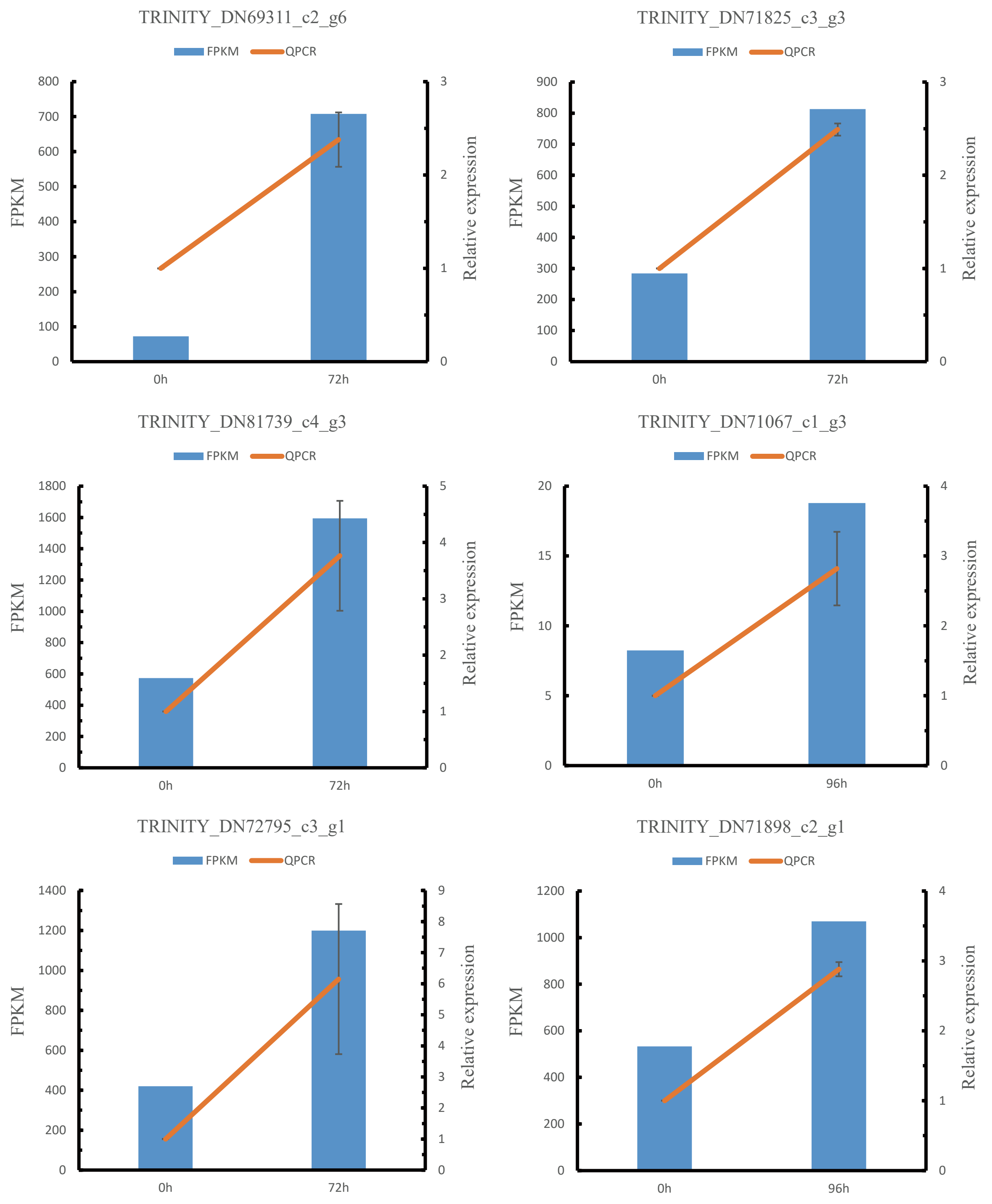

Supplementary Figure 7 | The expression levels of selected DEGs.

The correlations between the expression profiles of the selected DEGs were determined by RNA-Seq and qPCR analysis. The left y-axes show FPKM values determined by RNA-Seq, and the right y-axes show relative expression levels determined by qPCR.

(**A**)

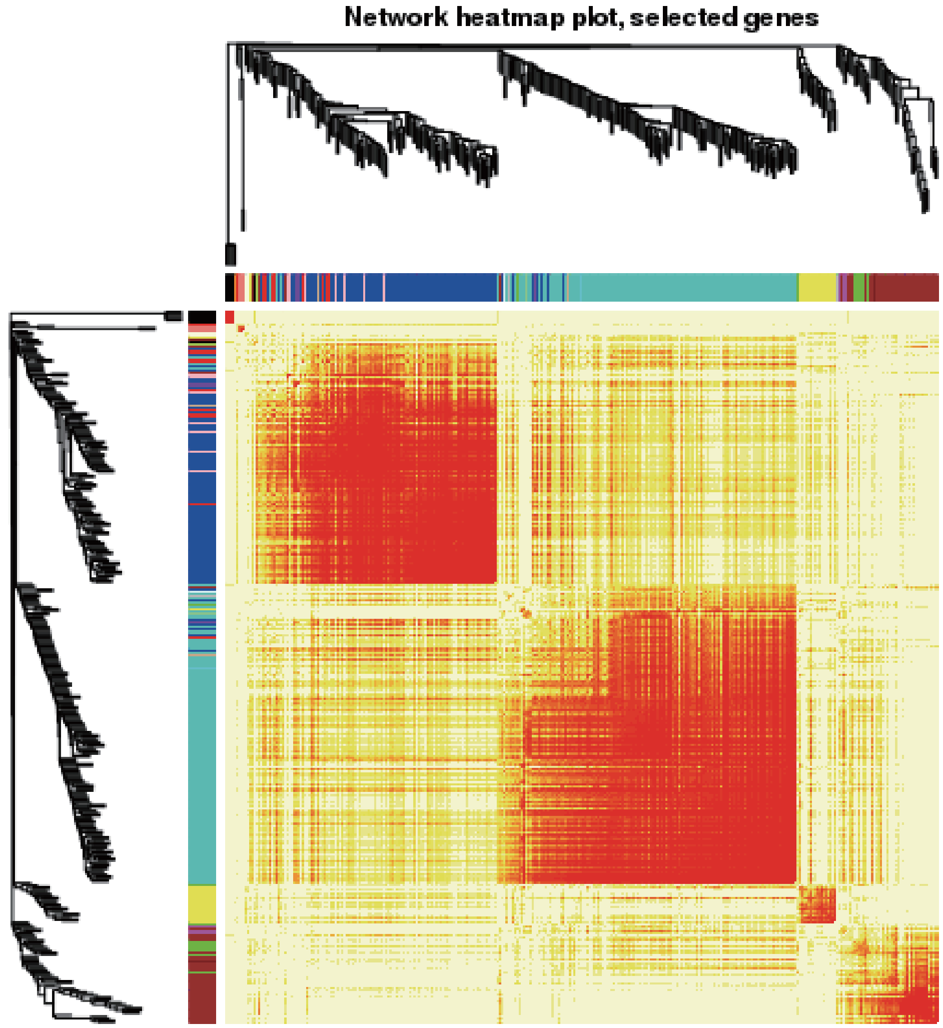

(**B**)

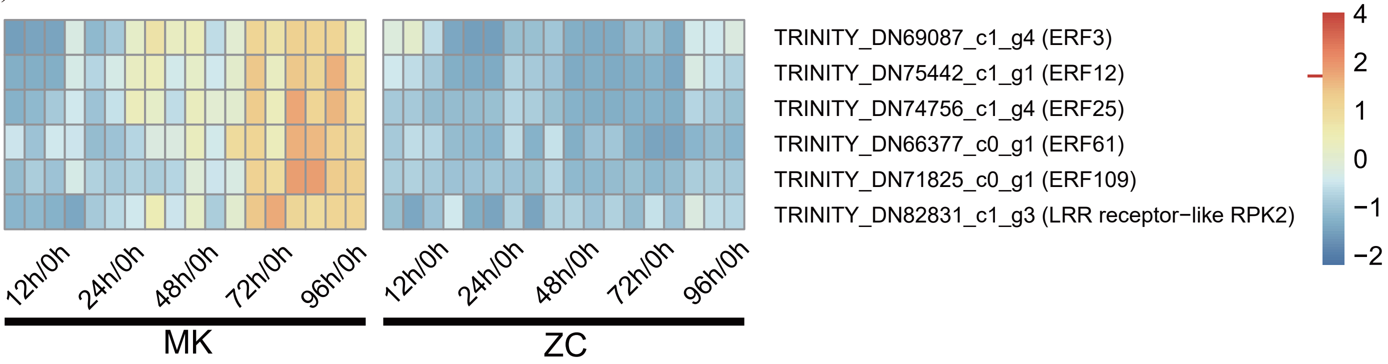

Supplementary Figure 8 | WGCNA of transcriptome data

(**A**) Dendrogram of genes based on coexpression network analysis of MK and ZC.

(**B**) Expression levels of the hub genes in the yellow module.

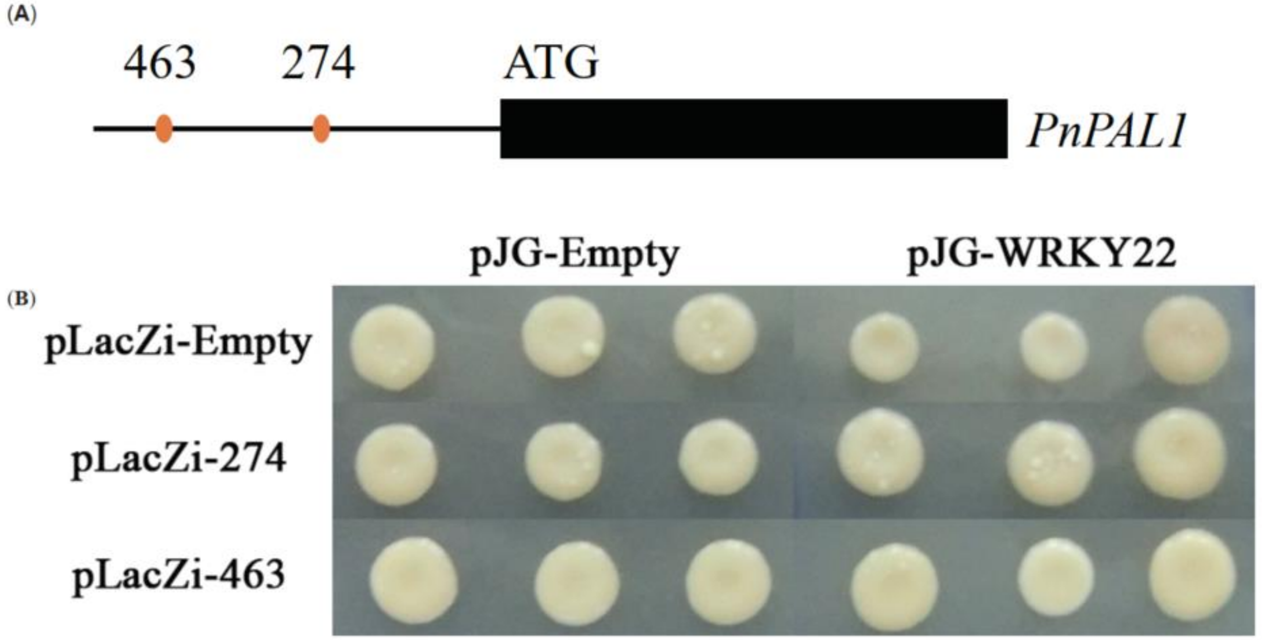

Supplementary Figure 9 | PnWRKY22 cannot bind to W-box cis-element in the *PnPAL1* promoter

(**A**) Location of the W-box cis-element in the *PnPAL1* promoter.

(**B**) Yeast one-hybrid assay revealing that PnWRKY22 cannot bind to the W-box cis-element in the *PnPAL1* promoter.

Supplementary Table 1 | Primers used in this study.

Supplementary Table 2 | Protein sequences used in this study.

Supplementary Table 3 | Comparison of six versions of *P. notoginseng* genome.

Supplementary Table 4 | BUSCOs in the *P. notoginseng* genome.

Supplementary Table 5 | Number of noncoding RNAs in the genome.

Supplementary Table 6 | Resistance genes identified in the *P. notoginseng* genome and the expression.

Supplementary Table 7 | Summary of RNA sequencing data.

Supplementary Table 8 | Pearson's correlation between samples.

Supplementary Table 9 | DEGs used for STEM and K-means cluster.
